# The Immediate Sterilisation of Infected Excreta

**Published:** 1900-11-17

**Authors:** 


### THE IMMEDIATE STERILISATION OF INFECTED EXCRETA.

What is evident from the account given by Dr. Howard Tooth is that unless the very greatest care be taken to sterilise all suspicious excreta, perhaps all excreta whether suspected or not of infection, from the very beginning before they have an opportunity of being disseminated by the agencies he mentions, the control of enteric fever by any of the means ordinarily employed must become a very difficult if not impossible task; hence we turn with much interest to another paper in the same issue of the *British Medical Journal* in which Major Cummins, R.A.M.C., describes how he set to work to insure the immediate sterilisation of the excreta from the enteric patients in the No. 1 Model School Military Hospital at Pretoria. He got a large 30-gallon iron "jackpot," into which a couple of gallons of carbolic solution (1 in 20) was first poured, and then the excreta. The pot was kept boiling day and night, the contents being frequently in part removed, but sufficient being always left to heat up at once the next stool emptied. Carbolic solution was added as required. In this way the practically instantaneous sterilisation of each stool the moment it left the bed-pan was secured both day and night. There was practically no smell beyond that of carbolic acid when the lid was raised. Not only were the stools from the enteric cases so treated, but all stools and urine from patients confined to bed and all the sweepings of the wards were emptied into the pot. Civil Surgeon A. M. Dodd, who was attached to the hospital, examined the contents of the pot bacteriologically and found it sterile, and so far as the experience of this hospital was concerned the process certainly seemed effectual, for no case of enteric fever appeared among the orderlies employed in nursing and in pioneer work. The average strength of these was about 35. It may perhaps be as well to note in regard to this method that the experience of some of the American surgeons, gained at the time of the war in Cuba, was to much the same effect, namely, that so great was the difficulty of controlling the spread of infection by the ordinary means, partly in consequence of the impossibility of isolating the ambulant cases sufficiently early, that it was strongly urged that the excreta of the whole camp, healthy as well as sick, should be received in galvanised iron troughs containing milk of lime, and then be either disinfected by heat or chemical means, or deeply buried before the material had an opportunity of drying and being distributed either as dust or by flies.

